# Screening and Identification of the Main Metabolites of Schisantherin a In Vivo and In Vitro by Using UHPLC-Q-TOF-MS/MS

**DOI:** 10.3390/molecules25020258

**Published:** 2020-01-08

**Authors:** Wei Feng, Ling-Yu Zhou, Rui-Feng Mu, Le Gao, Bing-Yuan Xu, Ming-Liang Liu, Li-Ying Niu, Xin-Guo Wang

**Affiliations:** School of Pharmaceutical Sciences, Hebei TCM Formula Granule Technology Innovation Center & TCM Formula Granule Research Center of Hebei Province University, Hebei University of Chinese Medicine, Shijiazhuang 050091, China; weifeng@hebcm.edu.cn (W.F.); zhuangxzz@163.com (L.-Y.Z.); maggiemu1986@163.com (R.-F.M.); ggaole@163.com (L.G.); xubingyuan77@163.com (B.-Y.X.); lml15133161856@163.com (M.-L.L.)

**Keywords:** schisantherin A, metabolites, UHPLC-Q-TOF-MS/MS, identification, multiple mass defect filter, multiple data processing

## Abstract

Schisantherin A is an active ingredient originating from *Schisandra chinensis* (Turcz.) which has hepatoprotective and anti-oxidation activities. In this study, in vitro metabolisms investigated on rat liver microsomes (RLMs) and in vivo metabolisms explored on male Sprague Dawley rats of Schisantherin A were tested, respectively. The metabolites of Schisantherin A were identified using ultra-high-performance liquid chromatography coupled with hybrid triple quadrupole time-of-flight mass spectrometry (UHPLC-Q-TOF-MS/MS). Based on the method, 60 metabolites were successfully identified and structurally characterized including 48 phase-I and 12 phase-II metabolites. Among the metabolites, 45 metabolites were reported for the first time. Moreover, 56 and eight metabolites were detected in urine and bile and 19 metabolites were identified in rats’ plasma. It demonstrated that hepatic and extra-hepatic metabolic pathways were both involved in Schisantherin A biotransformation in rats. Five in vitro metabolites were structurally characterized for the first time. The results indicated that the metabolic pathways mainly include oxidation, reduction, methylation, and conjugation with glucuronide, taurine, glucose, and glutathione groups. This study provides a practical strategy for rapidly screening and identifying metabolites, and the results provide basic data for future pharmacological and toxicology studies of Schisantherin A and other lignin ingredients.

## 1. Introduction

**Figure 1:**
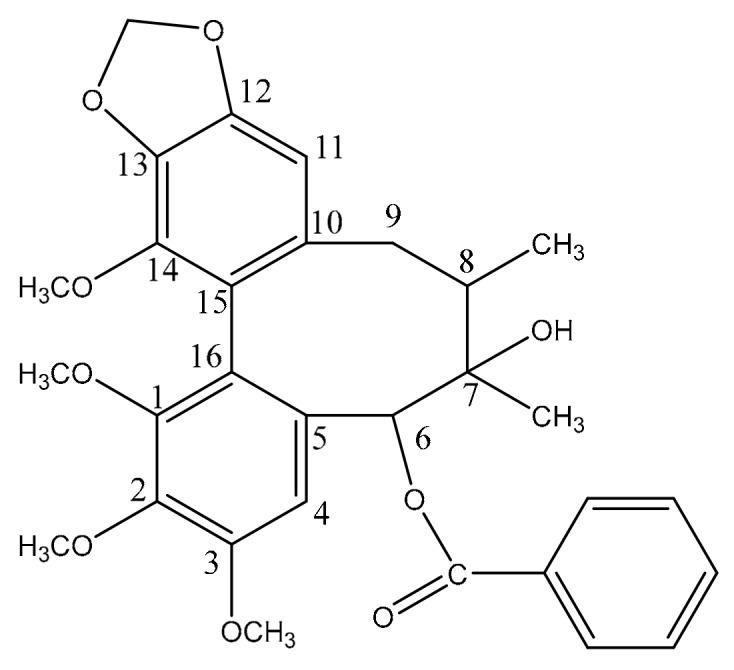
Chemical structure of Schisantherin A.

The traditional Chinese medicine *Schisandra chinensis* (commonly named as WuWeiZi in Chinese), and its dried mature fruits (Turcz.) Baill or *Schisandra sphenanthera Rehd* et Wils, belong to a type of herbal ingredient, and has been used for thousands of years [1,2]. The main components in *Schisandra chinensis* contain lignans, trierpenoids, proanthocyanidins, organic acids and fatty acids, volatile oils, and sugar [3,4,5,6]. Modern pharmacological and clinical studies have shown that *Schisandra chinensis* possesses hepatoprotective effects, anti-oxidant, anti-tumor, anti-human immunodeficiency virus (HIV), antiviral activity, and antagonistic activity towards platelet-activating factors [7,8,9,10,11]. Schisantherin A (the structure is shown in Figure 1) is a lignans compound of the highest concentration in *Schisandra chinensis* [12,13,14], and has the effects of protecting the liver, anti-oxidant, anti-tumor, etc. [15,16,17]. Recently, studies have analyzed Schisantherin A in rat biological samples after oral administration of an extract from Wuweizi [18,19] and herbal preparations, such as Shen-Song-Yang-Xin capsule, which contains the medicinal material of Wuweizi [20]. The metabolism of Schisantherin A has been reported, however, it has only detected a few metabolites [21,22]. In recent studies, metabolite identification is a part of drug discovery and development, and metabolism in vivo plays an important role in explaining and predicting efficacy and toxicity [23].

Recently, ultra-high performance liquid chromatography coupled with hybrid triple quadrupole time-of-flight mass spectrometry (UHPLC-Q-TOF-MS/MS) combined with pattern recognition analysis has become a robust and unbiased discrimination method that is used to identify the constituents of biological samples [24,25]. In order to detect as many metabolites as possible, a novel and practical multiple mass defect filter (MMDF) technique is used to identify drug metabolites according to different bio-transformations that have distinct mass defect values. Thus, minor metabolites are monitored and the MS/MS spectra are obtained in a single injection cycle [26,27]. On the other hand, off-line data processing technologies, such as mass defect filter (MDF), product ion filter (PIF), neutral loss filter (NLF), extract ion chromatogram (XIC), and isotope pattern filter provided by Metabolite-Pilot^TM^ and Peakview^TM^ software (AB Sciex) also play an important role in the identification of the complex compounds and metabolites [28,29,30].

**Figure 2:**
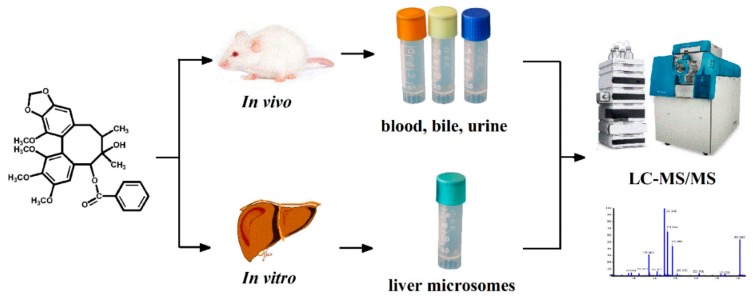
Overview of the experimental design.

In this research contribution, a rapid and ingenious approach known as UHPLC-Q-TOF-MS/MS combining pattern recognition analysis is employed to rapidly screen and characterize metabolites of Schisantherin A in vivo and in vitro. The overview of the experimental design is shown in Figure 2. Sixty metabolites in vivo and in vitro are achieved by the UHPLC-Q-TOF-MS/MS analysis based on the MS/MS spectra and Clog P values. Five metabolites were detected in rat liver microsomes (RLMs). In addition, the metabolic pathways of Schisantherin A are summarized. These results can help understand the metabolism mechanism of Schisantherin A.

## 2. Results

In this study, a total of 60 metabolites of Schisantherin A were detected, including 48 phase-I and 12 phase-II metabolites. Among the metabolites, 56 and eight metabolites were detected in urine and bile and 19 metabolites were identified in rats’ plasma. Five metabolites were detected in RLMs.

### 2.1. Analytical Strategy and Metabolite Analysis

In this study, the strategy of metabolite identification was developed based on a Triple TOF instrument with on-line data acquisition in the light of MMDF, dynamic background subtraction (DBS) dependent, and multiple post data-mining technologies. The analytical strategy consisted of four steps [31,32]: (1) On-line data acquisition is the first step. By using a unique and novel MMDF-and DBS-dependent data acquisition method, the full mass scan was performed, and accurate MS/MS data sets were obtained. The ability of identifying the drug-related MS/MS ions among the background and matrix-related MS/MS ions was promoted through the application of DBS. Therefore, the lower-level drug metabolite scan is clearly captured. (2) For the post-acquisition data processing, the AB software provides various data processing tools, such as XIC, MDF, PIF, and NLF. By using the XIC and MDF methods, the molecular weights and the elemental compositions of metabolites derived from the accurate mass measurements are readily predicted. In order to extract the precursors from the accurate MS/MS data, PIF and NLF techniques were applied. (3) The structures of the metabolites of Schisantherin A were clarified based on the accurate mass measurement, relevant drug bio-transformation knowledge, previously investigated fragmentation patterns of Schisantherin A, and MS/MS spectra of metabolites. (4) Distinguishing the isomers is the last step. Many metabolites have isomers because of the structure of Schisantherin A. Clog P, an important parameter, was used to estimate the retention time of the isomers. Clog P is the *log P* value calculated by the program Clog P (Chemdraw Ultra 14.0). Generally, the compounds with a larger Clog P value commonly have a longer retention time in a reversed phase liquid chromatography system.

### 2.2. Structural Assignment of the Product Ions of Schisantherin A

**Figure 3:**
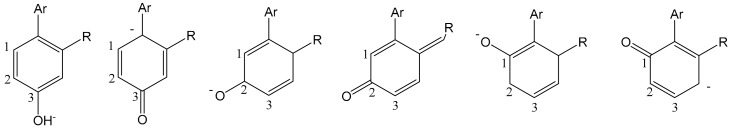
Resonant structures of Schisantherin A after the methyl group was eliminated.

Schisantherin A has a structure with methylenedioxy group, methyl groups, methoxy groups, benzoxyl group, and hydoxyl group. Thus, the neutral loss of C_2_H_2_O_2_, benzoic acid, H_2_O, methyl and methoxy groups are the major fragmentation pathways by collision-induced dissociation. As for loss of methyl groups and methoxy groups, the stability of the carbon ions was studied. The intermediates were more easily formed due to the enhanced stability of the carbon ion. There are four possible scenarios existing for the elimination of the methyl group: 1-CH_3_, 2-CH_3_, 3-CH_3_, and 14-CH_3_, and when the -CH_3_ was eliminated, two resonant structures were formed, respectively (Figure 3). When 3-CH_3_ was eliminated, the two resonant structures with the aryl group at the counterpoint of the 3-position made the carbanion more stable; when 2-CH_3_ was eliminated, the two structures made the whole conjugated system more stable; while when 1-CH_3_ or 14-CH_3_ were eliminated, the intermediate carbanion formation grew unstable. Therefore, the order of demethylation was: 3-CH_3_ > 2-CH_3_ > 1-CH_3_ = 14-CH_3_. However, for the elimination of the methoxy group, the case was totally different. Since the methylenedioxy group and methoxy group are electron-donating groups, they both increase the electron density of the adjacent position. Therefore, the susceptibility to demethoxylation was 2-OCH_3_ > 14-OCH_3_ > 1-OCH_3_ > 3-OCH_3_. For oxidation reactions, the order of the oxidation position was tertiary carbon > secondary carbon > primary carbon.

**Figure 4:**
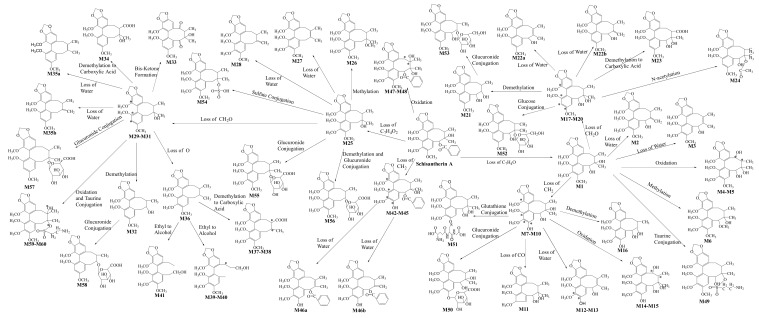
Proposed major metabolic pathway of Schisantherin A in a rat model.

The retention time of Schisantherin A was 11.99 min under the chromatographic condition, and a deprotonated molecular ion [M + Na]^+^ was formed at *m*/*z* 559.1948 in a positive scan mode. Based on the structure of Schisantherin A, there is a benzoic acid substituent at C-6, so it is easy to lose a molecule of benzoic acid to form the ion at *m*/*z* 415. There is also a hydroxyl group at C-7, so it is easy to lose a molecule of H_2_O to form the ion at *m*/*z* 397. Then after the loss of C_6_H_5_COOH, if the groups HCHO, OCH_3_, C_3_H_6_, and CH_3_CHO were successfully lost, the ions at *m*/*z* 385, 384, 373, and 371 were formed, respectively. After subsequently losing OCH_3_ from the ion *m*/*z* 397, the fragment ion *m*/*z* 366 was formed; while after losing the OCH_3_ from the ion *m*/*z* 373, the fragment ion *m*/*z* 342 was formed. When the OHC_3_ and CH_3_ groups were eliminated successfully from the ion *m*/*z* 371, the product ions *m*/*z* 356, 340, and 325 were formed. According to the high-resolution mass spectral information, the proposed major metabolic pathway of Schisantherin A in rats was shown in Figure 4. The information of molecular formulas, retention times, and fragment ions for the 60 metabolites are summarized in Table S1.

### 2.3. Identification of Phase-I Metabolites In Vivo

#### 2.3.1. Loss of C_7_H_4_O and Oxidation Reactions

M12–M13: The mass spectra of M12–M13, which were detected at the retention time of 7.75 and 8.37 min, gave molecule ions [M + H]^+^ at *m*/*z* 401.1584 and 401.1613 (C_22_H_24_O_7_), respectively. The quasi-molecular ions of M12 and M13, 18 Da lower than the quasi-molecular ions of M7–M10, suggest the loss of a H_2_O group. Based on the structure of Schisantherin A, this ion was formed due to the presence of an OH group at C-7. Then the protonated molecule formed a double bond between C-7 and C-8 through a rearrangement process at the dibenzocyclooctadiene structure. According to the demethylation rule, 3-CH_3_ and 2-CH_3_ were easily lost, for which the two compounds M12–M13 were speculated. As the Clog P values (Table S1) of the two structures were different, they were tentatively identified. M12 was tentatively identified as 7,8-dehydration-6-debenzoyl-3-demethylation-Schisantherin A and M13 was identified as 7,8-dehydration-6-debenzoyl-2-demethylation-Schisantherin A.

M14–M15: The deprotonated molecule ions [M + H]^+^ at *m*/*z* 435.1635 and 435.1642 were 16 Da higher than that of M7–M10, which implies that oxidation came up after Schisantherin A lost C_7_H_4_O and CH_2_ groups. The fragment ions at *m*/*z* 417, 399, 386, and 368 were formed by the loss of H_2_O, 2H_2_O, H_2_O and OCH_3_, 2H_2_O, and OCH_3_, respectively. Based on the structure of Schisantherin A and the demethylation rule, the oxidation reaction may occur at C-8 or C-9 after loss of 3-CH_2_. As the Clog P values of M14 and M15 were not equal, they were distinguished clearly.

There were more such metabolites like M1–M5, M7–M11, M16–M24. M5, M7, M9–M18, and M20–M24 were newly detected metabolites.

#### 2.3.2. Loss of C_7_H_4_O_2_ and Oxidation Reactions

M37–M38: M37 and M38, showing the molecular ion [M + H]^+^ at *m*/*z* 431.1696 and 431.1682, were eluted at 4.78 and 5.08 min respectively, which was 30 Da higher than that of M36. The diagnostic product ion at *m*/*z* 339 was observed by the loss of 46 Da, which suggests that a molecular HCOOH was lost from a protonated molecular ion. Other fragment ions at *m*/*z* 416, 401, 400, 385, and 369 were observed by loss of CH_3_ and OCH_3_ individual or combined. According to the fragment interpretation, the elemental formula was assigned as C_23_H_26_O_8_ by the loss of C_7_H_4_O_2_, O, and demethylation to carboxylic acid. The demethylation to carboxylic acid reaction usually occurred at 7-position and 8-position, and after 7-CH_3_ or 8-CH_3_ was lost, the carboxyl oxidation occurred at the demethylation position. Since the Clog P values of M37 and M38 were identical, they were indistinguishable.

M39–M41: Three metabolites M39–M41, eluted at 4.23, 5.1 and 5.31 min, respectively, were detected in the extracted ion chromatogram from *m*/*z* 389.1587 to 389.1594. The quasi-molecular ion of each of them was 12 Da less than that of M36. The distinctive product ion at *m*/*z* 371 ([M + H − H_2_O]^+^) was observed in the MS/MS spectra of M39–M41, so it is determined that M39–M40 were ethyl to alcohol metabolites of Schisantherin A after loss of C_6_H_5_COOH and O groups. The fragment ion at *m*/*z* 330 was detected in the MS/MS spectrum of M39 and M40 by the loss of C_3_H_5_ from the product ion at *m*/*z* 371, which showed that the OH group was connected to the 8-position carbon. While, in the MS/MS spectrum of M41, the typical fragment ions at *m*/*z* 344 ([M + H − H_2_O − C_2_H_3_]^+^) and 342 ([M + H − H_2_O − C_2_H_5_]^+^) were observed, which indicated that the OH group was connected with 7-position carbon. Because the Clog P values of M39 and M40 were equal, they were indistinguishable. The M41 had a special Clog P value, so it was identified reasonably.

There were more such metabolites like M25–M36. M30–M35 and M37–M41 were newly detected metabolites.

#### 2.3.3. Loss of CH_2_ and Oxidation Reactions

M46: M46 exhibited the deprotonated molecule ion [M + H]^+^ at *m*/*z* 505.1843, which was 18 Da less than that of M42–M45, eluted at 5.94 min under the experiment conditions. A host of diagnostic fragment ions were obtained at *m*/*z* 477, 399, and 383 by loss of CO, C_7_H_6_O_2_, and C_7_H_6_O. The metabolite M46 was inferred as C_29_H_28_O_8_, and was formed when the CH_2_ and H_2_O groups were lost from Schisantherin A. When the H_2_O group is lost, a double bond may form between C-7 and C-17 or C-7 and C-8. Thus, there were two compounds of M46. Since the two compounds had equal Clog P values, the location was not determined.

There were more such metabolites like M42–M45. M45–M46 were newly detected metabolites.

#### 2.3.4. Oxidation Reactions

M47–M48: M47 and M48 were eluted at 6.88 and 7.74 min with the deprotonated molecule ion [M + H]^+^ from *m*/*z* 553.2051 to 553.2068, which was 16 Da higher than that of Schisantherin A. It was suggested that oxidation reaction occurred for Schisantherin A. Then considering the structure of Schisantherin A, the oxidation reaction may occur at 8-position or 9-position. The Clog P values of the two compounds were unequal, so they were distinguished. They were both newly detected metabolites.

### 2.4. Identification of Phase-II Metabolites In Vivo

#### 2.4.1. Lost C_7_H_4_O and Phase-II Reactions

M49: M49, showing the molecule ion [M + H]^+^ at *m*/*z* 540.1908, was eluted at 2.66 min, which was 123 Da higher than that of M1. The major fragment ions at *m*/*z* 522, 433, 415, and 347 were observed by loss of H_2_O, C_2_H_5_NSO_2_, and C_5_H_10_O. The loss of 107 Da indicated that M49 was a taurine conjugation product, and the fragment ion at *m*/*z* 347 implied that a C_7_H_4_O group was lost from Schisantherin A. Therefore, M49 was inferred as C_25_H_33_NO_10_S.

There were more such metabolites like M50–M53. Five metabolites of this type were all newly detected metabolites.

#### 2.4.2. Lost C_7_H_4_O_2_ and Phase-II Reactions

M54: M54, eluted at 2.99 min, had the deprotonated molecule ion [M + H]^+^ at *m*/*z* 497.1461, and it was 80 Da higher than that of M25. The MS/MS data was obtained at *m*/*z* 417 [M + H − SO_3_]^+^, and 331 [M + H – SO_3_ − C_5_H_10_O]^+^. According to the information, it was obvious that Schisantherin A lost C_7_H_6_O_2_ and that sulfate conjugation reaction occurred to the hydroxyl group at C-7. Hence, the formula of M54 was deducted as C_23_H_28_O_10_S.

M55: M55, eluted at 2.65 min, showed deprotonated molecule ion [M + H]^+^ at *m*/*z* 593.2223, and it was 176 Da higher than that of M25. The typical fragment ions were found at *m*/*z* 417 [M + H − C_6_H_8_O_6_]^+^, 399 [M + H − C_6_H_8_O_6_ − H_2_O]^+^, and 331 [M + H − C_6_H_8_O_6_ − C_5_H_10_O]^+^. On the basis of the information, it was indicated that the glucuronide conjugation reaction occurred to the hydroxyl group at C-7 after Schisantherin A lost C_7_H_6_O_2_ group_._ Therefore, M55 was inferred as C_29_H_36_O_13_.

There were more such metabolites like M56–M60. Seven metabolites of this type were all newly detected metabolites.

### 2.5. Identification of Metabolites In Vitro

In this study, five phase-I metabolites were detected. M11, M25, M36, and M44 were detected both in vivo and in vitro, while M6 was not detected in vivo. The identification of M6 was shown as follows. M6: In the MS/MS spectrum, M6, eluted at 4.46 min, displayed the deprotonated molecule ion [M + H]^+^ at *m*/*z* 447.2007, 14 Da more than the deprotonated M1, which implied that methylation reaction occurred after the loss of C_7_H_4_O. The fragment ion at *m*/*z* 429 was formed when H_2_O was lost from the ion *m*/*z* 447, which indicated that the methylation reaction occurred at C-6 OH. The protonated molecular ion mainly displayed product ions at *m*/*z* 416, 401, 386, and 370 via the individual or combined loss of CH_3_ or OCH_3_. According to the composition shift (loss of C_7_H_4_O + methylation) given by the metabolite software, it was deduced that M6 was 6-debenzoyl-6-methylation-Schisantherin A.

Descriptions of other metabolites’ detection/identification are provided in Supplementary Materials.

## 3. Materials and Methods

### 3.1. Chemicals and Materials

Schisantherin A was purchased from the National Institute for Food and Drug Control (Beijing, China), and the purity was over 98% according to the HPLC analysis. Acetonitrile, ammonium acetate, and acetic acid were used for chromatography (HPLC grade; Fisher, Fair Lawn, NJ, USA). Water was purified by a Milli-Q academic purification system (Millipore, ELIX100, Bedford, MA, USA). Analytical grade carboxymethyl cellulose sodium (CMC-Na) was purchased from Kermel Chemical Reagents Development Centre (Kermel, Tianjin, China). β- NADPH(β-nicotinamide adenine dinucleotide 20-phosphate), glucose-6-phosphate, glucose-6-phosphatedehydrogenase, and UDPGA (uridine-50-diphosphoglucuronic acid trisodium salt) were acquired from Sigma Chemical Co. (St. Louis, MO, USA). Alamethicin was purchased from J&K Scientific (Beijing, China). Tris-HCl was obtained from Phygene Life Sciences Co. Ltd. (Fuzhou, China). MgCl_2_ was from Tianjin Hongyan Chemical Reagent Factory (Tianjin, China). RLMs were laboratory-made at the Department of Pharmaceutical Analysis in the School of Pharmacy, Hebei Medical University, and stored at −80 °C until used.

### 3.2. Standard Solutions Preparation

Schisantherin A was dissolved in 100% methanol at the exact concentration of 0.5 mg/mL.

### 3.3. Preparation of Liver Microsomes

The preparation of rat liver microsomes was assessed by employing the previously published method [33]. The fresh and weighted livers were perfused with 0.9% ice-cold saline and homogenized with 0.05 mol/L Tris-HCl buffer containing 0.25 mol/L sucrose (pH = 7.4). The mixture was centrifuged at 20,000 *g* for 30 min in a low temperature environment, and the supernatant was collected. The collected supernatant was centrifuged once more at 100,000 *g* for 60 min under the same conditions, and the precipitate was washed with cold Tris-HCl solution after removing the supernatant. The precipitate was resuspended with Tris-HCl solution to prepare the liver microsomes after centrifuging the mixture at 100,000 *g* for 60 min. Ultimately, the Lowry method was adopted to measure the protein concentration of the liver microsomes.

### 3.4. Microsomal Incubation

**Phase I metabolism:** A typical incubation mixture (200 μL final volume) was carried out in a 1 mol/L phosphate buffer (pH 7.4) containing rat microsomes (1.0 mg/mL), 3.3 mmol/L MgCl_2_, 1.3 mmol/L β-NADPH, 3.3 mmol/L glucose-6-phosphate, 1.0 U/mL glucose-6-phosphate dehydrogenase, and 0.5 mg/mL Schisantherin A methanol solution (1% of final volume). Pre-incubation was performed at 37 °C for 5 min before adding NADPH to start the reaction. Then, NADPH was added to the phase I reactions, and the reactions were incubated for 1 h at 37 °C and were terminated by the addition of 1 mL cold methanol. The mixture was extracted by vortex-mixing for 5 min and then centrifuged at 12,000 rpm for 10 min. The organic phase was collected and evaporated under nitrogen gas. The residues were reconstituted in 100% methanol (500 µL). The supernatant was filtered through a 0.22 mm polytetrafluoroethylene (PTFE) membrane filter and stored at −20 °C until analyzed. The control sample was incubated without an NADPH generating system, followed by the same treatment, while the blank sample was incubated without Schisantherin A.

**Phase II metabolism:** The incubation mixture (200 μL final volume) containing 1.0 mg/mL rat liver microsomes, 2 mmol/L UDPGA, 3.3 mmol/L MgCl_2_, 25 μg/mL alamethicin in 1 mol/L Tris-HCl buffer (pH 7.4) and 0.5 mg/mL Schisantherin A was pre-incubated for 5 min at 37 °C before adding UDPGA to start the reaction. Then, UDPGA was added to the phase II reactions, and the reactions were incubated for 1 h at 37 °C and were terminated by the addition of 1 mL cold methanol. The mixture was extracted by vortex-mixing for 5 min and then centrifuged at 12,000 rpm for 10 min. The upper solution was collected by nitrogen evaporation, after adding 500 µL to dissolve residual solids. The supernatant was filtered through a 0.22 mm PTFE membrane filter and stored at −20 °C until being analyzed. The control sample was incubated without UDPGA following the same treatment, while the blank sample was incubated without Schisantherin A.

### 3.5. Animals and Drug Administration

All experiments on rats were conducted in accordance with the guidelines of the Committee on the Care and Use of Laboratory Animals in our laboratory. Ethical approval for the study was granted by the Ethics Committee of Hebei University of Chinese Medicine (ethical no. DWLL2018021). Eighteen male Sprague Dawley rats (250–280 g) were obtained by the Experimental Animals Center of Hebei Medical University. The rats were randomly divided into 6 groups with 3 rats per group (groups 1, 3, 5, the blank sample group of plasma, urine, bile, respectively; groups 2, 4, 6, the experimental sample group of plasma, urine, bile, respectively). All rats were kept in an environmentally controlled breeding room with a temperature of 22 ± 2 °C, relative humidity 50 ± 5%, 12 h dark/light cycle for 7 days before the experiments were performed. During this time the rats had free access to filtered tap water and a standard animal diet. The rats were fasted for 12 h before the experiments but with free access to filtered tap water. Schisantherin A powder was suspended in 0.5% CMC-Na solution and was orally administered to 9 rats of the medication groups 2, 4, and 6 at a dose of 20 mg/kg body weight. The blank groups (groups 1, 3, and 5) were given equivalent CMC-Na solution without Schisantherin A powder. All animals were sacrificed immediately following the samples collection.

### 3.6. Biological Samples Collection

Plasma samples (groups 1 and 2) collection: The blood of the rats was taken from the eye canthus at 0.5, 1, 2, 4, and 6 h after administration, respectively. The blood samples were centrifuged at 12,000 rpm for 5 min, and the supernatant was collected and then combined to obtain the mixture plasma samples.

Urine samples (groups 3 and 4) collection: The rats were housed in separate metabolic cages with free access to filtered tap water. The urine samples were collected over a 0–24 h period after oral administration of CMC-Na solution with Schisantherin A powder or without Schisantherin A powder, respectively.

Bile samples (groups 5 and 6) collection: The rats were immediately anesthetized with urethane (at the dose of 1.0 g/kg) by intraperitoneal injection and fixed on a wooden plate after administered by oral gavage. Then the common bile ducts were cannulated with an EP-10 tube (ID = 0.07 cm) to collect the bile samples from 0 to 24 h. All samples were kept at −80 °C.

### 3.7. Samples Pretreatment

For plasma samples, 3 mL methanol were added to an aliquot of 600 μL plasma, and vortexed for 5 min. The mixture was centrifuged at 12,000 rpm for 10 min, and then the supernatant was separated and evaporated to dryness under a stream of nitrogen gas at 35 °C. Then the residue was reconstituted with 100 μL of methanol, vortexed for 1 min, then centrifuged at 12,000 rpm for 10 min, and the supernatant was separated. An aliquot of 4 μL was injected into the UHPLC-ESI(electrospray ionization) -Q-TOF system for analysis.

SPE (solid phase extraction) procedures (Waters Sep-Pak^®^ Vac 3cc, 200 mg C_18_ Cartridges) were used for urine and bile sample pretreatment, and each sample was extracted independently. A total of 5 mL methanol and 5 mL distilled water was used to precondition the SPE cartridge. Each sample was vortexed for 30 s and centrifuged at 12,000 rpm for 10 min before loaded into the SPE cartridge. Then, the SPE cartridge was washed with 2 mL water and eluted with 2 mL methanol. The eluent was evaporated to dryness under a stream of nitrogen gas at 35 °C. Then the residue was reconstituted with 500 μL of methanol, vortexed for 1 min, then centrifuged at 12,000 rpm for 10 min, and the supernatant was separated. An aliquot of 4 μL was injected into the UHPLC-ESI-Q-TOF system for analysis.

### 3.8. Instrumentation and Measurement Conditions

UHPLC-ESI-Q-TOF-MS/MS analysis was performed on an Agilent UPLC system (Agilent Technologies, Santa Clara, CA, USA), which was coupled to a hybrid quadrupole time-of-flight tandem mass spectrometer equipped with Turbo V sources and a Turbo ion spray interface (AB SCIEX Triple-TOF 5600+) (AB SCIEX, Redwood City, CA, USA). A Thermo Accucore C18 column (150 mm × 2.1 mm, 2.6 μm) held at 40 °C was used for chromatographic separation. The flow rate was 0.4 mL/min and the sample injection volume was 4 μL. The mobile phase consisted of water containing 0.1% acetic acid, 0.1 mmoL ammonium acetate (A), and acetonitrile (B) which was delivered using a linear gradient program as follows: 10%–40% B from 0 to 5 min, 40%–75% B from 5 to 25 min, 70%–100% B from 25 to 28 min.

The Q-TOF-MS system with an ESI source was performed in a positive ion electrospray mode. The optimized parameters were set as follows: ion spray voltage, 5.5 kV; turbo spray temperature, 550 °C; DP (declustering potential), 60 V. Nitrogen was used as the nebulizer and the auxiliary gas. Furthermore, the nebulizer gas (gas 1), heater gas (gas 2), and curtain gas were set to 55, 55, and 35 psi, respectively. The CE (collision energy) was set at 45 eV, and the CES (collision energy spread) was 15 eV, enabling us to obtain an average EPI scan spectrum when the CE was 30, 45, and 60 eV. A full scan was run in the positive mode with a mass range from *m*/*z* 100 to 1000 amu and with a 200-ms accumulation time. The IDA (information dependent acquisition) was used to trigger the acquisition of MS/MS spectra for ions matching the IDA criteria. As MMDF and DBS (dynamic background subtraction) were used to screen the profile and perform a production scan to avoid the omission of minor metabolites, and used to trigger the IDA function in the experiment. In addition, auto calibration delivery system (CDS) regulated MS and MS/MS automatically. For data acquisition, the Analyst^®^ TF 1.6 software (AB SCIEX, Los Angeles, CA, USA) was used.

## 4. Discussion

Many medicinal plants contain chemical compounds, such as lignan, that exhibit antioxidant properties and hepatoprotective effects. Schisandra is the dried mature fruits of *Schisandra chinensis*, which is widely applied in folk medicine of the Russian Far East, Korea, Japan, and northeastern China, and is often considered to be an example of a medicinal plant used as a sedative and tonic agent in TCM (traditional Chinese medicine). Previous phytochemical studies showed that *Schisandra* is a rich source of biphenyl cyclooctene lignans, characterized by Schisantherin A, Schisantherin B, Schisanhenol, Deoxyschisandrin, Schisandrin B, and Schisandrin C as the main constituents responsible for treating disease [18]. Biphenyl cyclooctene lignans present in the plant species under study possess a unique structure, and their biotransformation has not been elucidated in detail. Studies of the metabolic pathways of Schisantherin A have been reported in China [21,22], but only 12 metabolites were detected. However, in this study, 60 metabolites were detected in vivo and in vitro. The metabolites for the biphenyl cyclooctene lignans were easily traced based on the fragmentation pathways of the known lignan standards and knowledge of biotransformations. The identification of drug metabolites, which may have intrinsic pharmacological activity, has become an integral part of the drug discovery and development process. On the basis of systematical characterization of the metabolites of Schisantherin A, we could gain a better understanding about the in vivo and in vitro metabolic processes of Schisantherin A. These results provide a solid basis for further pharmacological and pharmacokinetic study of Schisantherin A, and will be helpful to reveal the action mechanism of Schisantherin A.

## 5. Conclusions

In the study, in vitro (rat liver microsomes) and in vivo (plasma, urine, and bile in rats) metabolic profiles were investigated by UPLC-Q-TOF-MS/MS. Consequently, a total of 60 metabolites were screened and characterized. To the best of our knowledge, 45 metabolites were reported for the first time. In addition, among the 60 metabolites, 48 were phase-I and 12 were phase-II metabolites, indicating that the main metabolic method of Schisantherin A is phase-I. Five in vitro metabolisms were concentrated in phase-I, thus, it was suggested that the metabolism pathway in vivo is more complicated than that of in vitro. Furthermore, 56 and eight metabolites were detected in urine and bile, while the other 19 and five metabolites were identified in rats’ plasma and liver microsomes, respectively, indicating that hepatic and extra-hepatic metabolic pathways were both involved in Schisantherin A biotransformation in rats. The methoxy group and the biphenyl cyclooctene were the metabolic sites indicated by the metabolite results. In addition, Schisantherin A mainly underwent oxidation and reduction reactions, which contained 48 phase-I metabolites. What is more, methylation, glucuronide, sulfate, taurine, glutathione, and glucose conjugation metabolites were detected in the analysis of the sample. Loss of C_7_H_4_O and C_7_H_4_O_2_ was the primary metabolic step, followed by further transformations through oxidation, carboxylic acid reaction, loss of CH_2_ and CH_2_O groups, and conjugation with groups. The present study explored a powerful analytical strategy for fast screening and identification of the metabolites of Schisantherin A using MMDF- and DBS-dependent on-line data acquisition combined with multiple post acquisition data processing. On the basis of systematical characterization of metabolites, a better understanding about the metabolic processes of Schisantherin A could be obtained. Moreover, these results set the foundation for further pharmacology and toxicology research.

## Supplementary Materials

The following are available online, The extracted ion chromatograms, chemical structures, identification, and detailed data of the 60 metabolites, in addition to the MS/MS spectrum and fragmentation pathways of Schisantherin A (Figures S1 and S2, Document S1, Table S1, Figures S3 and S4) are available online, respectively.
